# Efficient bone regeneration of BMP9-stimulated human periodontal ligament stem cells (hPDLSCs) in decellularized bone matrix (DBM) constructs to model maxillofacial intrabony defect repair

**DOI:** 10.1186/s13287-022-03221-3

**Published:** 2022-12-27

**Authors:** Yuxin Zhang, Wenping Luo, Liwen Zheng, Jing Hu, Li Nie, Huan Zeng, Xi Tan, Yucan Jiang, Yeming Li, Tianyu Zhao, Zhuohui Yang, Tong-Chuan He, Hongmei Zhang

**Affiliations:** 1 Chongqing Key Laboratory for Oral Diseases and Biomedical Sciences, The Affiliated Hospital of Stomatology of Chongqing Medical University, 426 Songshibei Road, Chongqing, 401147 China; 2 Department of Pediatric Dentistry, The Affiliated Hospital of Stomatology, Chongqing Medical University, Chongqing, China; 3 Chongqing Municipal Key Laboratory of Oral Biomedical Engineering of Higher Education, Chongqing, China; 4 Molecular Oncology Laboratory, Department of Orthopaedic Surgery and Rehabilitation Medicine, The University of Chicago Medical Center, Chicago, IL 60637 USA

**Keywords:** Bone defect, Human dental mesenchymal stem cells (hDMSCs), BMP9, Decellularized bone matrix (DBM) construct, Intrabony defect repair model

## Abstract

**Background:**

BMP9-stimulated DPSCs, SCAPs and PDLSCs are effective candidates for repairing maxillofacial bone defects in tissue engineering, while the most suitable seed cell source among these three hDMSCs and the optimal combination of most suitable type of hDMSCs and BMP9 have rarely been explored. Moreover, the orthotopic maxillofacial bone defect model should be valuable but laborious and time-consuming to evaluate various candidates for bone regeneration. Thus, inspired from the maxillofacial bone defects and the traditional in vivo ectopic systems, we developed an intrabony defect repair model to recapitulate the healing events of orthotopic maxillofacial bone defect repair and further explore the optimized combinations of most suitable hDMSCs and BMP9 for bone defect repair based on this modified ectopic system.

**Methods:**

Intrabony defect repair model was developed by using decellularized bone matrix (DBM) constructs prepared from the cancellous part of porcine lumbar vertebral body. We implanted DBM constructs subcutaneously on the flank of each male NU/NU athymic nude mouse, followed by directly injecting the cell suspension of different combinations of hDMSCs and BMP9 into the central hollow area of the constructs 7 days later. Then, the quality of the bony mass, including bone volume fraction (BV/TV), radiographic density (in Hounsfield units (HU)) and the height of newly formed bone, was measured by micro-CT. Furthermore, the H&E staining and immunohistochemical staining were performed to exam new bone and new blood vessel formation in DBM constructs.

**Results:**

BMP9-stimulated periodontal ligament stem cells (PDLSCs) exhibited the most effective bone regeneration among the three types of hDMSCs in DBM constructs. Furthermore, an optimal dose of PDLSCs with a specific extent of BMP9 stimulation was confirmed for efficacious new bone and new blood vessel formation in DBM constructs.

**Conclusions:**

The reported intrabony defect repair model can be used to identify optimized combinations of suitable seed cells and biological factors for bone defect repair and subsequent development of efficacious bone tissue engineering therapies.

**Supplementary Information:**

The online version contains supplementary material available at 10.1186/s13287-022-03221-3.

## Background

The integrity of maxillofacial bone is critical to human oral health and has important supporting and protecting functions [1], although reconstruction and repair of severe bone defects caused by trauma or tumors remain major clinical challenges [2]. In these circumstances, the use of autografts and allografts are popular strategies in clinical treatment [3, 4]. However, the limited source, donor site morbidity, immune rejection and laborious procedures hamper bone graft applications [4–6]. Recently, the use of tissue engineering strategies represents a promising alternative approach [7, 8]. With the increased exploration of various seed cells and biological factors, which could be candidates for bone regeneration, the optimal combinations should be effectively determined for regenerative medicine.

Constructing the orthotopic defects in maxillofacial bone to evaluate various combinations of candidate factors for bone defect repair is the gold standard, while these studies could be time consuming and needed elaborate surgical procedures [9]. Ectopic bone formation systems, which is commonly in the way of directly injecting biofactor-stimulated cell suspensions subcutaneously on the flanks of mice, are considered alternative methods for evaluating bone tissue engineering in vivo since they are economical and easy to be carried out [10]. However, the use of traditional ectopic systems has limitations since they lack a bony environment, which is vital for bone remodeling process [9, 11]. Furthermore, the shapes of the newly formed bony masses could be irregular. Due to these aspects, the use of traditional ectopic systems may undermine the evaluation efficiency. Decellularized bone matrix (DBM) can supply a microenvironment similar to the in vivo bone defect [12]. Therefore, developing a modified ectopic system by DBM should provide an intrabony environment for evaluating bone tissue engineering therapies, which could also limit cell suspension within a controlled space.

The source of stem cells is wide-ranged, and different seed cell sources exhibit distinct outcomes in mesenchymal stem cells (MSCs)-based therapies. Human dental mesenchymal stem cells (hDMSCs) exhibit multiple lineage differentiation potential, including robust new bone and new dentin formation for tissue engineering [13, 14]. Thus, hDMSCs may be used as critical seed cells to support and promote maxillofacial bone defect repair. Dental pulp stem cells (DPSCs), periodontal ligament stem cells (PDLSCs) and apical papillary stem cells (SCAPs) are among the common dental stem cells reported to possess more clinical application prospects [15–17], as they can be obtained from distinct dental tissue sources and exhibit distinct biological characteristics and differentiation capacities [18]. Therefore, it is important to compare the osteogenic potential of these distinct sources of hDMSCs *in vivo* and to identify the most efficacious one for tissue engineering use.

Numerous biological factors have been demonstrated to augment the osteogenic differentiation activity of hDMSCs, which are also recognized as the rate-limiting step of effective tissue engineering [19]. Bone morphogenetic proteins (BMPs) have been demonstrated to play important roles in bone regeneration [20]. In our comprehensive analysis of 14 types of human BMPs, we demonstrated that BMP9 is one of the most potent factors that can induce osteogenic differentiation of mesenchymal stem cells (MSCs) and play critical roles in tooth and alveolar bone development although BMP9 is one of the least studied BMPs [21–26]. In recent years, the potent osteogenic capability of hDMSCs stimulated with BMP9 has been demonstrated both in vivo and in vitro [27]. However, the distinct types of hDMSCs may possess distinct osteogenic activity in response to the stimulation of BMP9. Besides, the dosage of stem cells required for efficacious bone regeneration may be reduced with the help of powerful biofactors. Thus, it is conceivable that the combination of hDMSCs and BMP9 can be further optimized for efficacious bone formation in tissue engineering applications.

In this study, we created an intrabony defect repair model by using decellularized bone matrix (DBM) constructs from the cancellous part of a porcine lumbar vertebral body with a defined bone structure to serve as modified ectopic systems, and then explored the optimal combination of suitable hDMSCs and BMP9 for effective bone regeneration in our fabricated constructs to model the repair of orthotopic maxillofacial bone defects.

## Methods

### Preparation of ring-shaped decellularized and demineralized bone matrix (rsDBM) disks

**Fig. 1:**
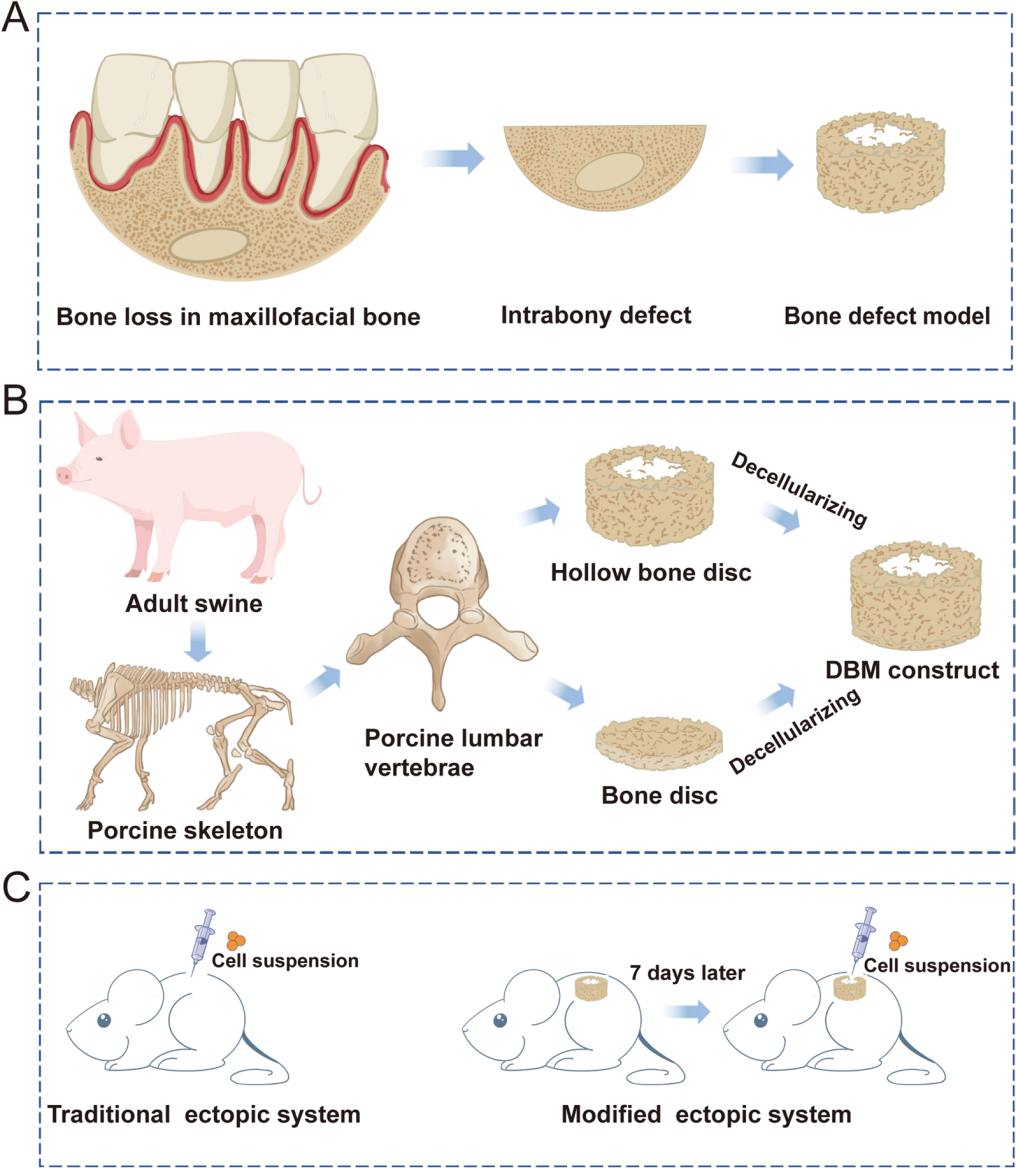
Design purpose and preparation process of the decellularized bone matrix (DBM) constructs. **A** Schematic representation of the proposed maxillofacial intrabony defect-inspired model using DBM. **B** Schematic depiction of the fabrication process of the DBM constructs. **C** Schematic representation of the modified ectopic system using fabricated DBM constructs

The rsDBM construct was prepared from adult porcine lumbar vertebral bodies (Fig. 1C). Briefly, normal and fresh lumbar vertebral tissue was obtained from a local slaughter house (Rongchang, Chongqing, China). After the remnant periosteum, muscle, and soft tissue were removed, the vertebral body was cut to 3 mm and 0.5 mm in thickness using a hard tissue microtome (Exakt 300 CP, Germany). After being washed with distilled water, the bone tissue was cut into either 5.7-mm-diameter and 3-mm-thick bone disks or 5.7-mm-diameter and 0.5-mm-thick disks from the middle region of the lumbar vertebral bone. The ring-shaped bone disks were further prepared from 5.7-mm-diameter and 3-mm-thick disks by removing the central 3 mm of bone tissue with a dental bur, resulting in hollow bone disks with a 5.7 mm outer diameter and 3 mm inner diameter. All bone disks were rinsed and soaked in PBS for 4 h at 4 °C, decellularized in 1% Triton X-100 for 12–24 h at 37 °C, degreased in 25 ml methanol at room temperature with gentle agitation and then soaked in 10 to 30 ml 100% ethanol for 4 h. Finally, the bone disks were rinsed with sterile PBS containing Pen-Strep and stored at − 80 °C until use.

### Fabrication and characterization of DBM constructs

A typical cylinder-shaped DBM construct used in this study was composed of two parts, a 3-mm-thick hollow bone disk and a 0.5-mm-thick “bottom” bone disk, which were glued together with a light curing dental bonding agent and light cured resin. The resulting hollow cylindrical container was measured with an outer diameter of 5.7 mm, inner diameter of 3 mm and overall thickness of 3.5 mm (Fig. 3B). The compression tests of the DBM constructs were conducted with a universal testing machine (C43.104, MTS, USA) with a constant deformation rate of 1 mm/min. The elastic modulus was calculated from the linear region of the stress–strain curve. The compressive strength was determined from the stress–strain curve using the 0.2% offset method. Three samples were tested in each group.

### Isolation and characterization of human dental mesenchymal stem cells (hDMSCs)

The use of human dental samples from clinical procedures was approved by the Ethics Committee of The Affiliated Hospital of Stomatology, Chongqing Medical University (CQHS-REC-2022 (LSNo.15)]. All subjects enrolled in the study signed the informed consent form. The methods of isolation and culture of dental stem cells were described previously [28–30]. Briefly, after the removal of teeth, periodontal ligament tissue was harvested with a sterile scalpel by scraping the middle one-third of the tooth root. Dental pulp tissue was collected after sectioning the crown using a dental drill, while apical papilla was obtained from the apical part of the dental papilla. All collected tissues were cut into small pieces and then digested with type I collagenase (Sigma, USA) for 30 min, followed by culturing in α-MEM (HyClone, New York, USA) containing 1% penicillin/streptomycin and 10% fetal bovine serum (FBS, HyClone, New York, USA) at 37 °C in 5% CO_2_. Culture medium was changed every three days until the primary cells migrated out of the tissue and reached confluence. Cells at passage 3 were used in the experiments. The expression of stem cell surface markers of hDMSCs was characterized by means of flow cytometry. The fluorescence-labeled antibodies were CD90-FITC (Sino Biological, China), CD29-FITC (Sino Biological, China) and CD45-FITC (Sino Biological, China) as previously described [31–33].

### Generation and amplification of recombinant adenoviruses expressing BMP9 and GFP

The HEK-293 or 293pTP cell line was used for adenovirus generation and amplification [34]. Recombinant adenoviruses expressing BMP9 (Ad-BMP9) and green fluorescent protein (Ad-GFP) were generated with AdEasy technology as previously reported [22, 23, 25]. Polybrene (10 µg/ml; Solarbio, Beijing, China) was applied to improve viral infection efficiency [35].

### Osteogenic induction

Primary hDMSCs were seeded in 10-cm cell culture dishes and infected with the indicated multiplicity of infection (MOI) of Ad-BMP9 or Ad-GFP. The GFP signal of the infected hDMSCs was detected under a fluorescence microscope (Carl Zeiss Microimaging GmbH, Gottingen, Germany) 24 h after infection. The GFP positivity of the hDMSCs was used as an indicator of the infection efficiency. The hDMSCs were harvested 24 h after infection for subcutaneous injection into DBM construct.

### RNA isolation and real-time quantitative PCR (RT-qPCR)

Total RNA was extracted from Ad-BMP9 or Ad-GFP infected SCAPs, DPSCs and PDLSCs using TRIzol (Invitrogen; Thermo Fisher Scientific, Inc.). 2 µg total RNA was transcribed into cDNA with the use of reverse transcription reaction kit (Invitrogen; Thermo Fisher Scientific, Inc.) in a volume of 20µL. The obtained cDNA samples were diluted three-fold with nuclease-free water. All RT-qPCR reactions were conducted using 2 × SYBR-Green qPCR Master Mix (Bimake, Houston, TX, USA). The qPCR cycles were 95 °C for 3 min, followed by 39 cycles at 95 °C for 10 s, 59 °C for 30 s, and one cycle at 95 °C for 5 s, 65–95 °C, incremented by 0.5 °C for 5 s. At the end of qPCR, the melting curve test was performed to validate the specificity of every primer pair. The *x* = 2^−ΔΔ*Ct*^ formula was used to calculate relative mRNA expression levels. Three technical replicates were utilized for every sample. *GAPDH* was used to be a reference gene. The primer sequences of the used genes are shown in Additional file 1: Table S1.

### Animal studies

All animal experiments were in compliance with the Animals (Scientific Procedures) Act (1986), and the approval of all procedures was granted by the Ethics Committee of The Affiliated Hospital of Stomatology, Chongqing Medical University [CQHS-REC-2022 (LSNo.15)]. Animal studies were divided into two parts, and a total of 25 specific pathogen-free (SPF) NU/NU athymic nude mice (6–8 weeks old, male, 20–25 g weight) were purchased from Beijing Vital River Laboratory Animal Technology Co., Ltd (11 mice for the first part of the experiment, followed by 14 mice after obtaining the result in the first section). The athymic nude mice were allowed three consecutive days for stabilization of physiological responses before the experiment began, and they were kept in ventilated cages at a temperature of 20℃ to 24℃ and maintained on a 12/12-h light/dark cycle with a maximum density of 5 mice per cage. All the surgeries were carried out in the dedicated laboratory and the mice were anesthetized with isoflurane (3–4% for anesthesia induces and 1–1.5% for anesthesia maintenance; 21–23% O_2_, balance N_2_), with two DBM constructs subcutaneously implanted on the flank of each mouse. A total of 48 DBM constructs were implanted and randomly divided into the following 16 groups (including 21 DBM constructs with 11 athymic nude mice in the first part of animal experiment and 27 DBM constructs with 14 athymic nude mice in the second part): The PBS/DBM construct (negative control group), GFP-DPSCs/DBM construct, BMP9-DPSCs/DBM construct, GFP-SCAPs/DBM construct, BMP9-SCAPs/DBM construct, GFP-PDLSCs/DBM construct, BMP9-PDLSCs/DBM construct, 1 × 10^6^ PDLSCs-BMP9 0.7/DBM construct, 1 × 10^6^ PDLSCs-BMP9 1.2/DBM construct, 1 × 10^6^ PDLSCs-BMP9 2.3/DBM construct, 2 × 10^6^ PDLSCs-BMP9 0.7/DBM construct, 2 × 10^6^ PDLSCs-BMP9 1.2/DBM construct, 2 × 10^6^ PDLSCs-BMP9 2.3/DBM construct, 3 × 10^6^ PDLSCs-BMP9 0.7/DBM construct, 3 × 10^6^ PDLSCs-BMP9 1.2/DBM construct, and 3 × 10^6^ PDLSCs-BMP9 2.3/DBM construct, were designed for the following experiments. GFP affected hDMSCs were used as the positive groups. Computer-based random-order generator was used to generate random numbers. The single DBM construct implanted on the flank of athymic nude mouse was the experimental unit and three experimental units were involved per group. A small sample size was selected for each group, since the use of our designed DBM construct was assessed in vivo the first time. Therefore, the present study enabled us gain basic evidence for using BMP9-hDMSC-DBM construct in more complex conditions. One week after implantation, the harvested cells suspended in 40 µl PBS were injected into the hollow area of the DBM constructs. All surgeries were done by the same surgeon during the period of 8:30am to 17:30 pm, and the experiment orders were randomized. The mice were monitored once daily for the wound healing condition after surgery, as well as the food and water intake, weight and activity. If the wound occurred severe suppurative infection or the weight of the mice decreased 20% compared to the weight before, the euthanasia should be used in advance. For each animal, investigators responsible for the data analysis were unaware of the group allocations, while the investigators responsible for the allocation and the surgery were aware of the group allocations, since the colors of pure PBS differed from the Ad-GFP or Ad-BMP9 affected cell suspensions in PBS.

### Micro-CT analysis

The animals were included in the study if they underwent successful DBM constructs implantation and injection treatment, defined by the almost healed conditions of wounds. The animals were excluded if severe inflammatory reaction occurred, or if the animal died prematurely, preventing the collection of Micro-CT and histological data. Included animals were killed with CO_2_ Euthanasia System (30–70% rate of the CO_2_ replacement per minute) 8 weeks post injection. The DBM constructs were harvested and fixed in 4% PBS buffered formalin (Solarbio; Beijing, China) for 2 days. The samples were scanned with micro-CT (Viva CT 40; Scanco Medical, Bassersdorf, Switzerland) at 70 kVp and 114 uA with a 15 μm voxel size. The acquired images were analyzed by using Mimics Research 19.0 software (Materialise, Lefen, Belgium) and 3-matic Research 11.0 software (Materialise, Lefen, Belgium) to evaluate newly formed bone, bone volume fraction (BV/TV), radiographic density (in Hounsfield units (HU)) and the height of newly formed bone. The two-dimensional (2D) images of the samples were reconstructed into three-dimensional (3D) models by importing the micro-CT DICOM dataset into Mimics Research 19.0 software (Materialise, Lefen, Belgium). The newly formed bone inside or outside the DBM constructs was also extracted for further analyses. Grayscale-based material properties were assigned to the volumetric mesh of new bone tissues based on Mimics Research 19.0 software (Materialise, Lefen, Belgium). The relationship between the Hounsfield units (HU) and new bone density related to porcine lumbar vertebrae was determined as previously described [36–38].

### Histologic evaluation and immunohistochemical staining

The harvested DBM constructs were fixed in 4% PBS buffered formalin, decalcified with EDTA solution and embedded in paraffin. The samples were serially sectioned. At least three sections from each sample were chosen for hematoxylin and eosin (H&E) staining. We also carried out trichrome staining on the selected sections. The staining results were recorded under a bright-field microscope (YC. YX—2050, Japan).

Immunohistochemical staining was conducted using the retrieved DBM construct sections. Rabbit anti-OCN (Servicebio, GB11233, 1:200), Rabbit anti-CD31 (Servicebio, GB113151, 1:600) antibody and rabbit anti-α-SMA (Servicebio, GB111364, 1:1000) antibody were used as the primary antibodies. The Streptavidin Peroxidase (SP) Kit (ZSGB-BIO, China) and diaminobenzidine (DAB) Peroxidase Substrate Kit (ZSGB-BIO, China) were used for the detection of staining. The absence of primary antibody groups was used as a negative control. The staining results were recorded under a bright-field microscope (YC. YX—2050, Japan). The acquired images were analyzed by using Image-Pro Plus 6.0 software (Media Cybernetics, USA) to evaluate Integrated Optical Density (IOD) for quantitative analysis.

### Statistical analysis

The quantitative data are presented as the mean ± SD, as all quantitative experiments were conducted in triplicate. Student’s t tests or one-way ANOVA tests performed with SPSS 21.0 were used to determine the statistical significance. The data normality test was carried out by SPSS 21.0 with the approach of normal probability plots to determine whether the data of the analysis were normally distributed and met the assumptions of the statistical approaches. *P* < 0.05 was considered statistically significant.

## Results

### The modified ectopic system DBM constructs can be reproducibly fabricated

**Fig. 2:**
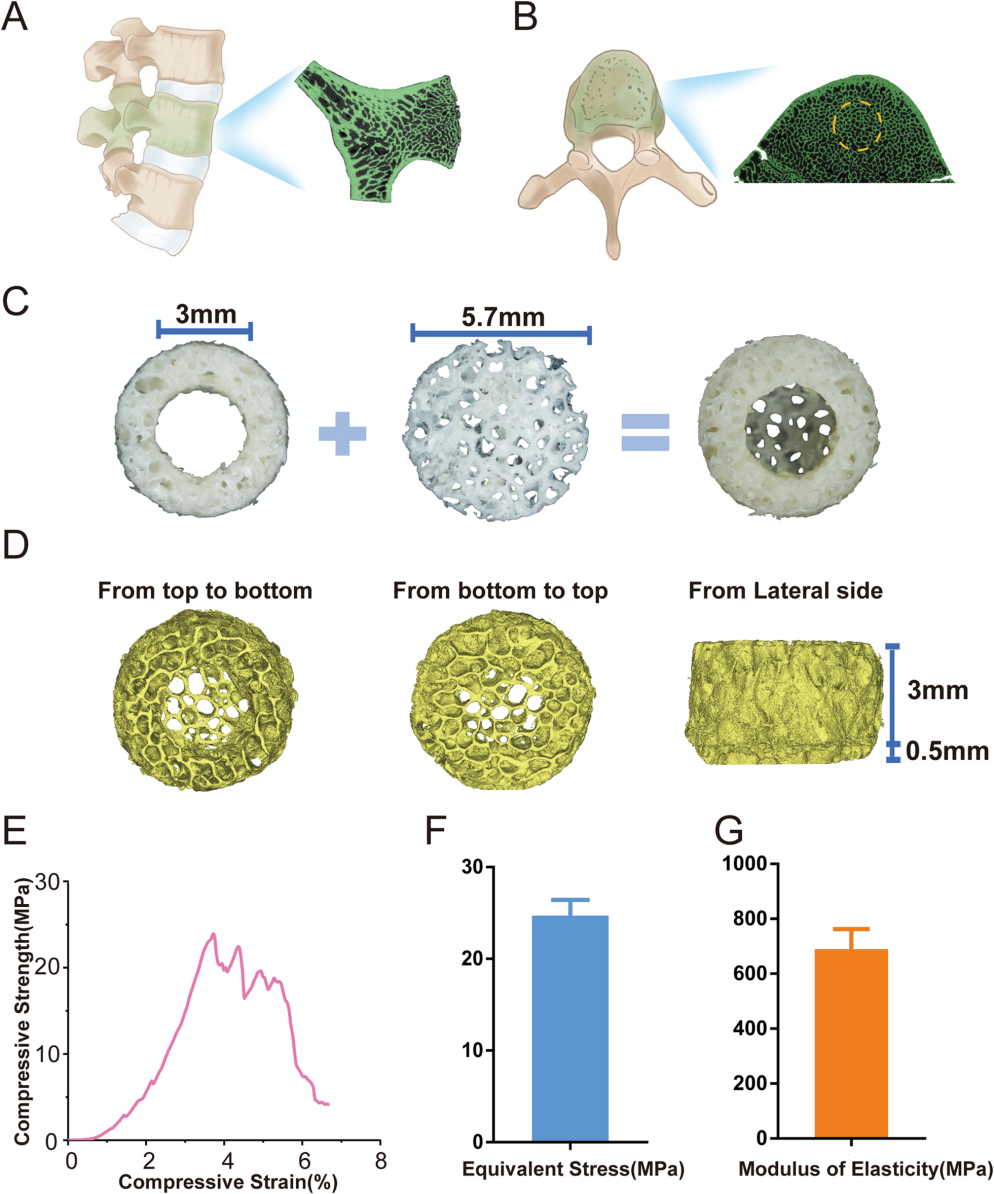
Characterization of the decellularized bone matrix (DBM) constructs. **A** Representative µCT images of porcine lumbar vertebrae bone at the vertical section. **B** A µCT image of a horizontal section of porcine lumbar vertebrae bone. The dotted circles in the image denote the selected area for DBM constructs. **C** Representative photographs of the processed DBM constructs. The 5.7-mm-outer diameter and 3-mm-inner diameter ring-shaped bone disk was stuck to the 5.7-mm-diameter bone disk to form a hollow construct. **D** Representative reconstructed 3D µCT images of the DBM constructs showing their porous structure. **E** DBM constructs were subjected to compression experiments in vitro, including stress–strain curve, equivalent stress (**F**) and modulus elasticity (**G**) of the DBM constructs in the compression test

To design a modified ectopic system, the maxillofacial intrabony defect-inspired model was utilized (Fig. 1A). Cylindrical-shaped DBM constructs with uniformly distributed trabecular pore properties were prepared from adult porcine lumbar vertebral body bone (Fig. 1B). Specifically, instead of directly injecting the cell suspension into the subcutaneous area, we designed a modified ectopic system that involved two procedures: first, subcutaneously implanting the DBM constructs, followed by injecting the cell suspension into the hollow area of the DBM constructs 7 days later (Fig. 1C). To assess the structural features of the trabecular bone disks, we scanned L4–L5 lumbar vertebrae samples with micro-CT (Fig. 2A, B). As shown in the radiographs, the selected area of the lumbar vertebrae for DBM construction exhibited a uniform porous structure (Fig. 2B).

To fabricate the DBM constructs, sectioned bone disks were subjected to decellularization, demineralization and digestion, resulting in a white, sponge-like structure (Fig. 2C). To provide adequate space for new bone regeneration, we designed a novel cylinder-shaped DBM construct, which appeared as a cylinder of 5.7 mm in outer diameter and 3 mm in inner diameter with a 0.5-mm-thick bone disk cemented to a 3-mm-thick hollow bone disk (Fig. 2C). Micro-CT imaging of the construct further confirmed the uniformity of the porous structure and the interconnected pores after decellularization (Fig. 2D).

Next, we measured the biomechanical characteristics of the DBM constructs and carried out compression tests; thus, stress–strain curves of the DBM construct were obtained (Fig. 2E). The construct showed an equivalent stress of 24.70 ± 1.73 MPa and a Young’s modulus of 689.53 ± 73.51 MPa, as demonstrated in Fig. 2F, G. These results indicated that the DBM construct exhibited sufficient compressive strength to support new bone regeneration and possessed a better cushioning capacity under stress.

### Primary DPSCs, SCAPs and PDLSCs stimulated by BMP9 are three convenient candidate cell sources for maxillofacial bone defect repair

**Fig. 3:**
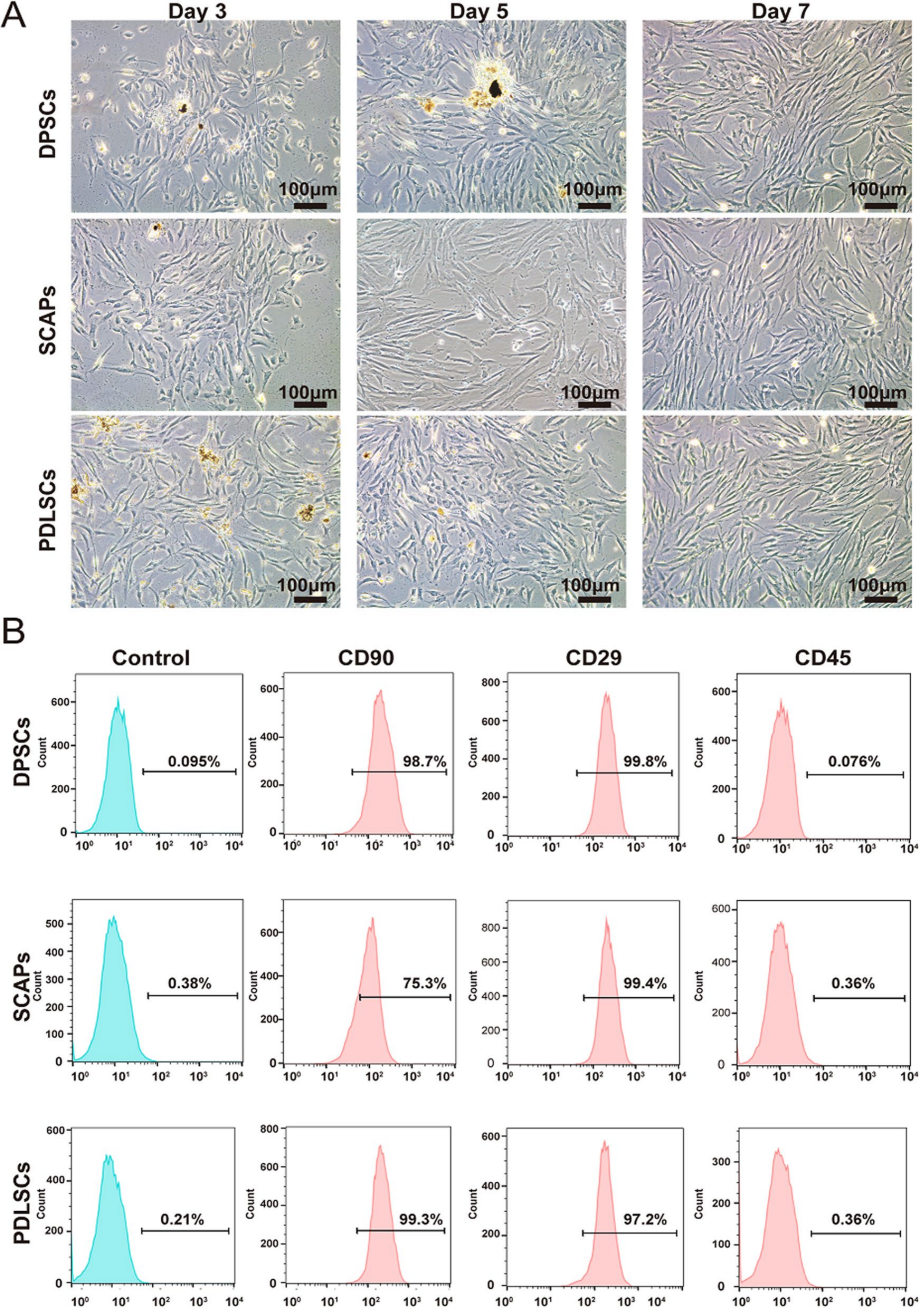
Isolation and characterization of primary human dental mesenchymal stem cells (hDMSCs). **A** Morphology of primary human DPSCs, SCAPs and PDLSCs. Primary human DPSCs, SCAPs and PDLSCs were isolated from different dental tissue sections and photographed at the indicated times (3, 5 and 7 days). Representative images are shown. **B** The expression of the mesenchymal stem cell surface markers CD90 and CD29 and the hematopoietic marker CD45 in isolated primary human DPSCs, SCAPs and PDLSCs was detected by flow cytometry

To evaluate the suitable cell sources for effective maxillofacial bone defect repair through our DBM constructs, which may recapitulate the bone regeneration events, BMP9-stimulated DPSCs, SCAPs and PDLSCs were chosen as candidate sources of osteoprogenitors. The bright-field images showed that primary hDMSCs grew well and appeared fibroblast-like fusiform (Fig. 3A). Flow cytometric analysis confirmed that the dental MSC markers CD90 and CD29 were highly expressed in these DMSCs, while the hematopoietic marker CD45 was negative (Fig. 3B), indicating that the isolated primary cells in this study possessed the characteristics of MSCs.

**Fig. 4:**
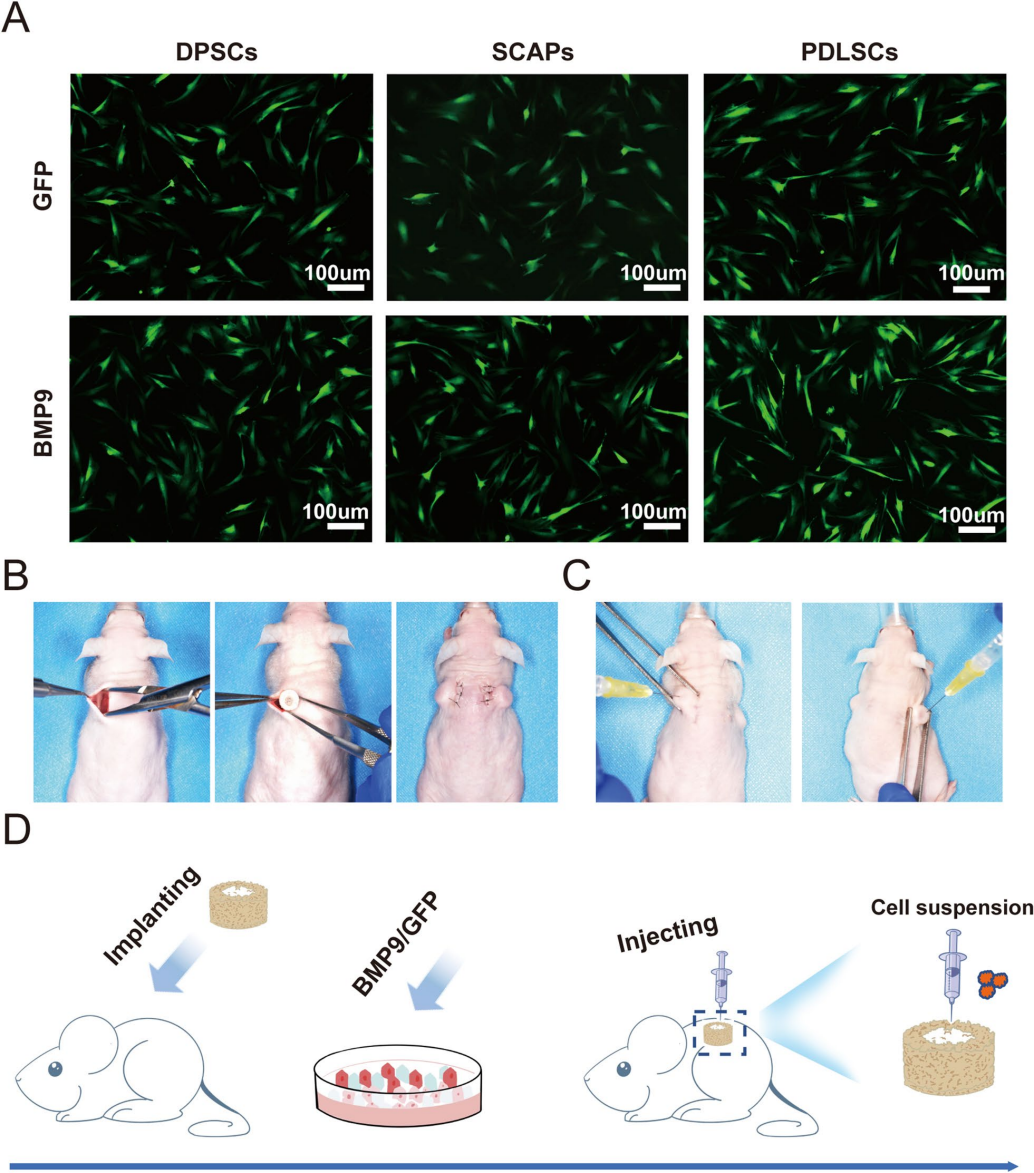
Cells were prepared and injected into the DBM constructs. **A** Human DPSCs, SCAPs and PDLSCs were infected with Ad-GFP and Ad-BMP9 for 1 d before collection. **B** Representative images showing the process of implanting DBM constructs into the subcutaneous area of athymic nude mice. **C** Photographs showing the process of injecting the infected hDMSCs into the DBM constructs 7 days after implantation. **D** Schematic illustration of the whole procedure in our modified ectopic system from implanting the construct to injecting the cell suspension

We next augmented the osteogenic ability of hDMSCs by infecting them with Ad-BMP9. GFP signals were detected in DPSCs, SCAPs and PDLSCs at 24 h after infection with Ad-BMP9 or Ad-GFP at an MOI of 1.4 (the dilution of the virus at which 75% of cells were infected) (Fig. 4A). To determine whether BMP9 induces the osteogenic activity of hDMSCs, the in vitro quantitative assays were carried out. We previously identified some significant early responsive target genes of BMP9 in MSCs like ID1, ID2 and CTGF [39–45]. We infected SCAPs, DPSCs and PDLSCs with Ad-GFP or Ad-BMP9 for 24 h, and the expression levels of ID1, ID2 and CTGF were significantly up-regulated when stimulated by BMP9 (Additional file 1: Fig. S1A). Furthermore, BMP9 was also demonstrated to induce the expression levels of osteogenic transcription factors RUNX2, osteopontin (OPN), alkaline phosphatase (ALP) and osterix (OSX) in hDMSCs at day 3 and/or day7 (Additional file 1: Fig. S1B). Following with this, the designed ectopic intrabony defect repair model was utilized to assess the osteogenic activity of these three BMP9-stimulated hDMSCs in vivo (Fig. 4B–D). Specifically, the plain DBM constructs (without cells) were subcutaneously implanted into athymic nude mice, with the hollow bone disk facing upward (Fig. 4B). After 7 days, the primary hDMSCs infected with Ad-BMP9 or Ad-GFP were subcutaneously injected into the hollow area of the implanted DBM construct (Fig. 4C).

### BMP9-stimulated PDLSCs induced the strongest bone formation efficiency among the three types of hDMSCs in the DBM constructs

**Fig. 5:**
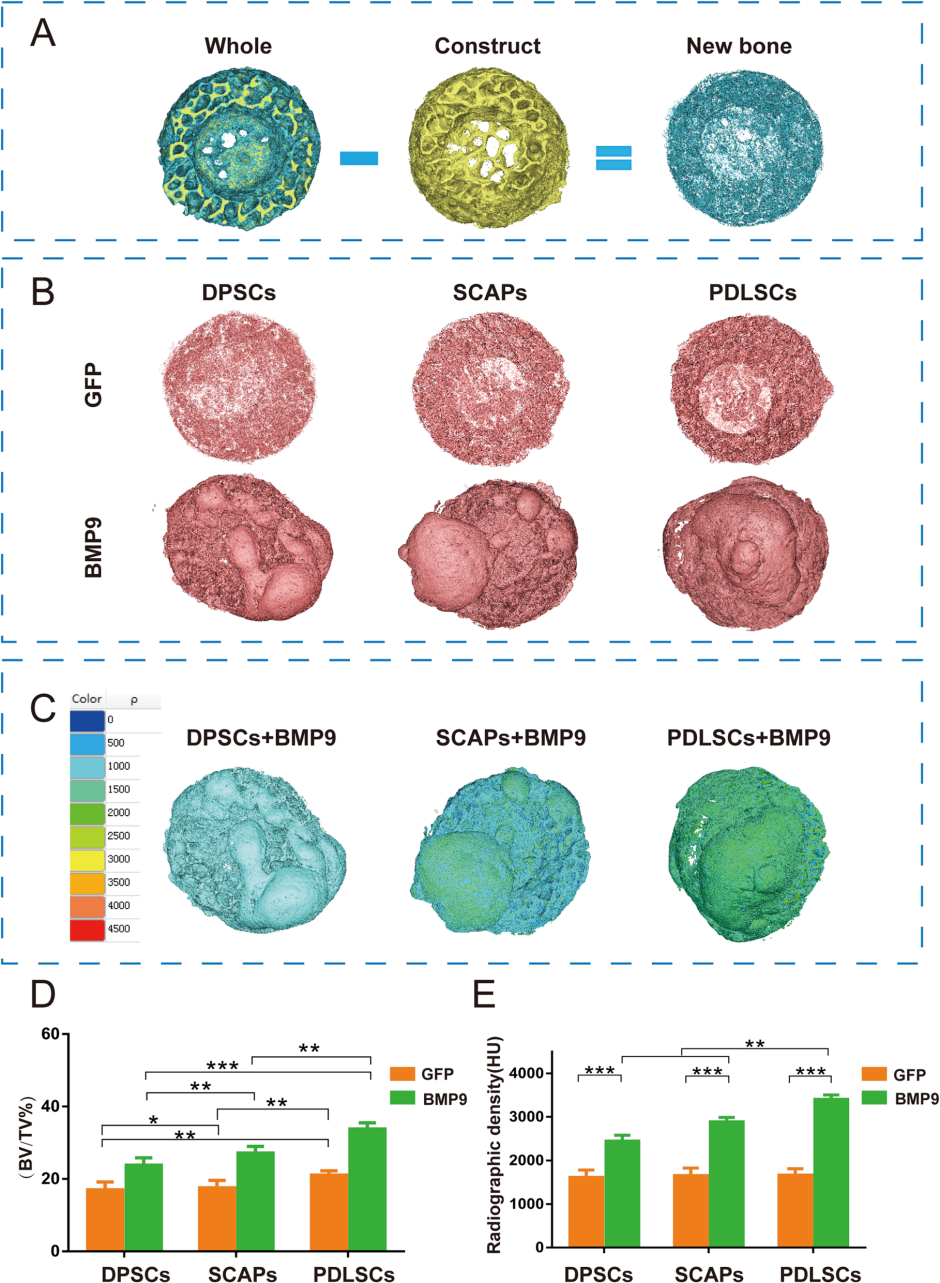
BMP9-stimulated PDLSCs exhibited the most potent osteogenic activity in DBM constructs. Eight weeks after the cell suspension injection, the animals were killed. Both the newly formed masses and DBM constructs were retrieved, fixed in formalin, and subjected to µCT imaging. **A** Schematic representation of the procedure for extracting new bone from the whole construct. **B** 3D reconstruction was performed for all scanned samples. Representative 3D µCT images of the new bone tissues that had separated from whole constructs (2 × 10^6^ cells-BMP9 1.4/DBM construct). **C** Representative density distribution images of the new bone separated from whole constructs. **D** and **E** The average new bone volume fraction (BV/TV) and radiographic density (in Hounsfield units (HU)) were analyzed using Mimics Research 19.0 software. All values are the means ± SDs; *P < 0.05, **P < 0.01, ***P < 0.001

To assess the evaluation efficiency of our modified ectopic system and screen for the most suitable type of hDMSCs stimulated by BMP9 for bone defect repair, we compared the osteogenic effect of these three distinct hDMSCs stimulated by BMP9 in our DBM constructs. The micro-CT imaging analysis revealed that significant new bone formation occurred inside the defect area of the DBM constructs. The net new bone tissues were extrapolated by subtracting original DBM constructs from the whole constructs based on the different thresholds of the constructs and the regenerated mineralized matrix (Fig. 5A). 3D reconstruction analysis of the newly formed bone revealed that the BMP9-PDLSCs/DBM construct group had a significantly larger volume of new bone than the other groups (Fig. 5B), suggesting that under the same conditions, BMP9-restored PDLSCs in the DBM constructs may yield the most robust new bone formation inside the defect. Further bone density distribution analysis of the micro-CT imaging reconstructions revealed that new bone densities varied significantly among the groups, while the BMP9-PDLSCs/DBM construct group exhibited the highest bone density among the three BMP9 treatment groups (Fig. 5C). We also analyzed the net new bone formation, new bone volume fraction (BV/TV) and radiographic density (in Hounsfield units (HU)) in the tested construct groups and found that while the BMP9-DPSCs/DBM and BMP9-SCAP/DBM construct groups filled their total volumes (i.e., BV/TV) by 24.47 ± 0.97% and 27.16 ± 0.85%, the BMP9-PDLSCs/DBM construct group’s BV/TV increased to 33.48 ± 1.29% (Fig. 5D, E). Consistent with the BV/TV results, the average HU in the BMP9-PDLSCs/DBM construct group was the highest among the three BMP9 treatment groups (Fig. 5D, E).

Further analysis of the sagittal plane of micro-CT imaging results for the retrieved bony masses revealed that the BMP9-DPSCs/DBM, BMP9-SCAP/DBM and BMP9-PDLSCs/DBM construct groups all possessed a newly formed bony mass that exceeded the inner space of the DBM constructs, indicating robust osteogenic activities of the hDMSCs in the presence of BMP9 (Additional file 1: Fig. S2A), which was further confirmed by the quantitative analysis of the new bone height based on micro-CT imaging data (Additional file 1: Fig. S3). Nonetheless, it is noteworthy that various degrees of new bone formation were observed in the PBS/DBM construct group, although the new bone deposition was limited to the inner edges of the construct, confirming the osteoinductive activity of the DBM constructs. Moreover, the DBM construct containing GFP-transduced hDMSCs showed increased osteogenic activity compared with the DBM construct without cells (Additional file 1: Fig. S4). Collectively, these results indicated that while the DBM construct itself was insufficient to initiate a robust bone healing program, the presence of DMSCs (especially PDLSCs) and BMP9 effectively promoted new bone formation in the modified ectopic system in vivo. Based on the results gained in this part, we selected PDLSCs as the most suitable seed cells among these three types of hDMSCs for the following experiments.

### An optimal dose of PDLSCs with a specific extent of BMP9 stimulation is adequate to promote efficacious new bone formation in DBM constructs

**Fig. 6:**
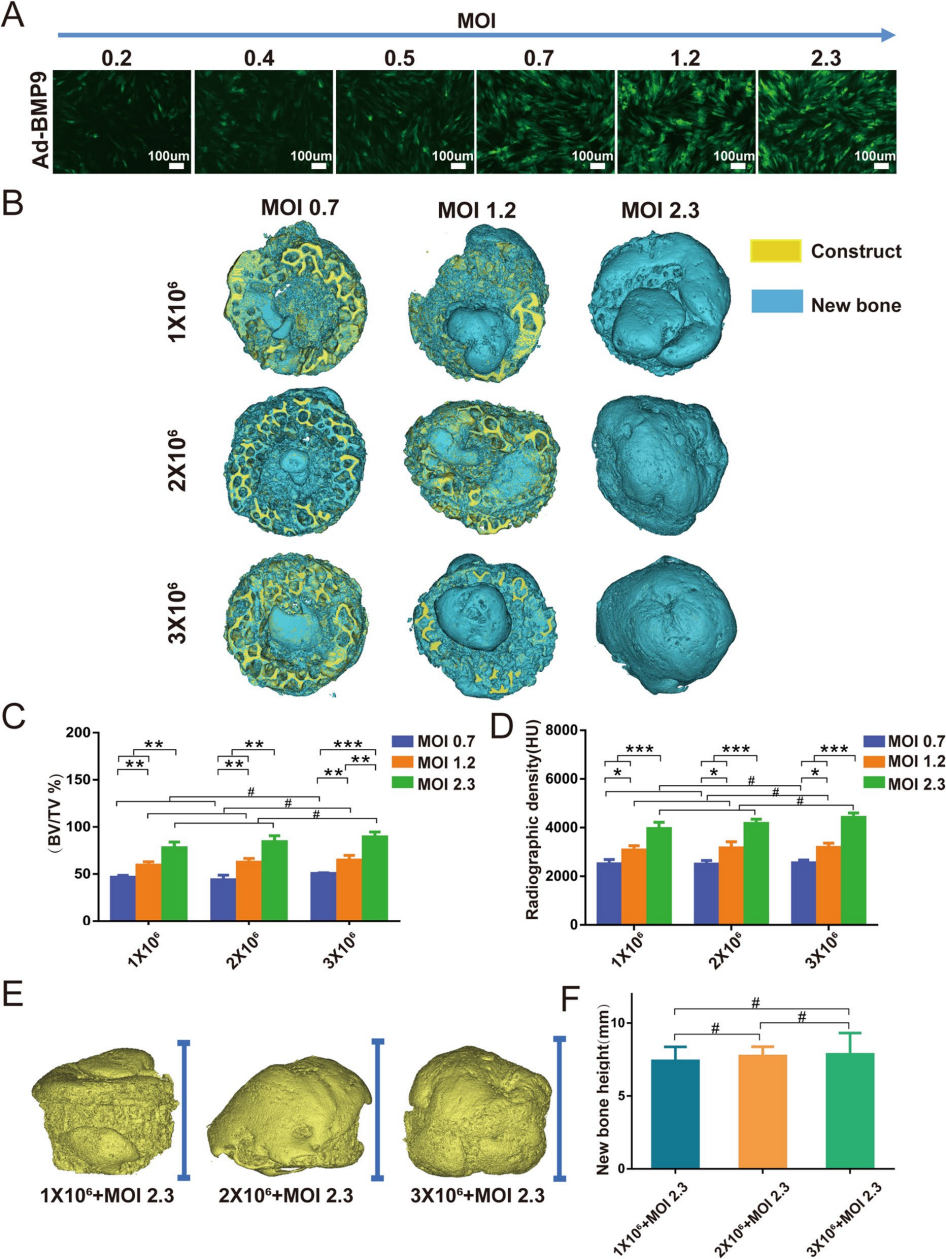
An optimal dose of BMP9-stimulated PDLSCs is adequate for bone formation in DBM constructs. **A** Representative fluorescent signals of PDLSCs with different MOIs of Ad-BMP9. Primary human PDLSCs were infected with Ad-BMP9 for 1 d. **B** The subcutaneously implanted DBM constructs loaded with infected cells were harvested at 8 weeks post injection, and 3D µCT images of the whole constructs were reconstructed. The yellow part is the construct, while the blue part is the newly formed bony mass. **C** and **D** The average BV/TV and radiographic density (in Hounsfield units (HU)) were analyzed using Mimics Research 19.0 software. **E** Reconstructed 3D µCT images showing the heights of new bone formation. **F** The heights of new bone were analyzed using Mimics Research 19.0 software. *P < 0.05, **P < 0.01, ***P < 0.001, # no statistical significance

To investigate whether the DBM construct can be used as a measuring tool to estimate approximate dose of stem cells for specific-sized bone defect repair, as well as explore the optimal combination of PDLSCs and BMP9, we infected PDLSCs at six different MOIs (0.2, 0.4, 0.5, 0.7, 1.2 and 2.3) without observing apparent cytotoxicity and chose MOIs of 0.7, 1.2 and 2.3 for the following experiments (Fig. 6A). The total cell numbers of PDLSCs varied from 1 × 10^6^/construct to 3 × 10^6^/construct, in which the cells were infected with Ad-BMP9 at MOIs of 0.7, 1.2 and 2.3 (Fig. 6A). After 8 weeks, mineralized projections inside the constructs were observed by micro-CT imaging, and representative 3D reconstructed images of the retrieved samples, including both regenerated mineralized matrix and intrabony defect constructs, showed new bone formation (Fig. 6B). As shown in 3D reconstructed images, the volumes of new bone were significantly augmented in a correlation with increased Ad-BMP9 MOIs, which did not exhibit obvious differences among groups with distinct cell concentrations but the same MOI (Fig. 6B). Quantitative analysis of micro-CT imaging data with the new bone volume fraction (BV/TV) and radiographic density (in Hounsfield units (HU)) further confirmed the results (Fig. 6C, D).

The retrieved bony masses from three different cell doses at the same Ad-BMP9 MOI of 2.3 nearly repaired the whole defect area inside the DBM constructs (Fig. 6B), and compared to BMP9 MOIs of 0.7 and 1.2 (Additional file 1: Fig. S2B), the new bone tissue exceeded the inner space of the DBM constructs (Fig. 6E). Quantitative analysis of the micro-CT images further revealed that there was no obvious difference in the new bone height among these three groups with distinct cell concentrations but the same MOI (2.3) (Fig. 6F). These results suggested that compared to cell doses of 2 × 10^6^/construct and 3 × 10^6^/construct, 1 × 10^6^/construct under BMP9 stimulation at an MOI of 2.3 was adequate to promote efficacious new bone formation.

**Fig. 7:**
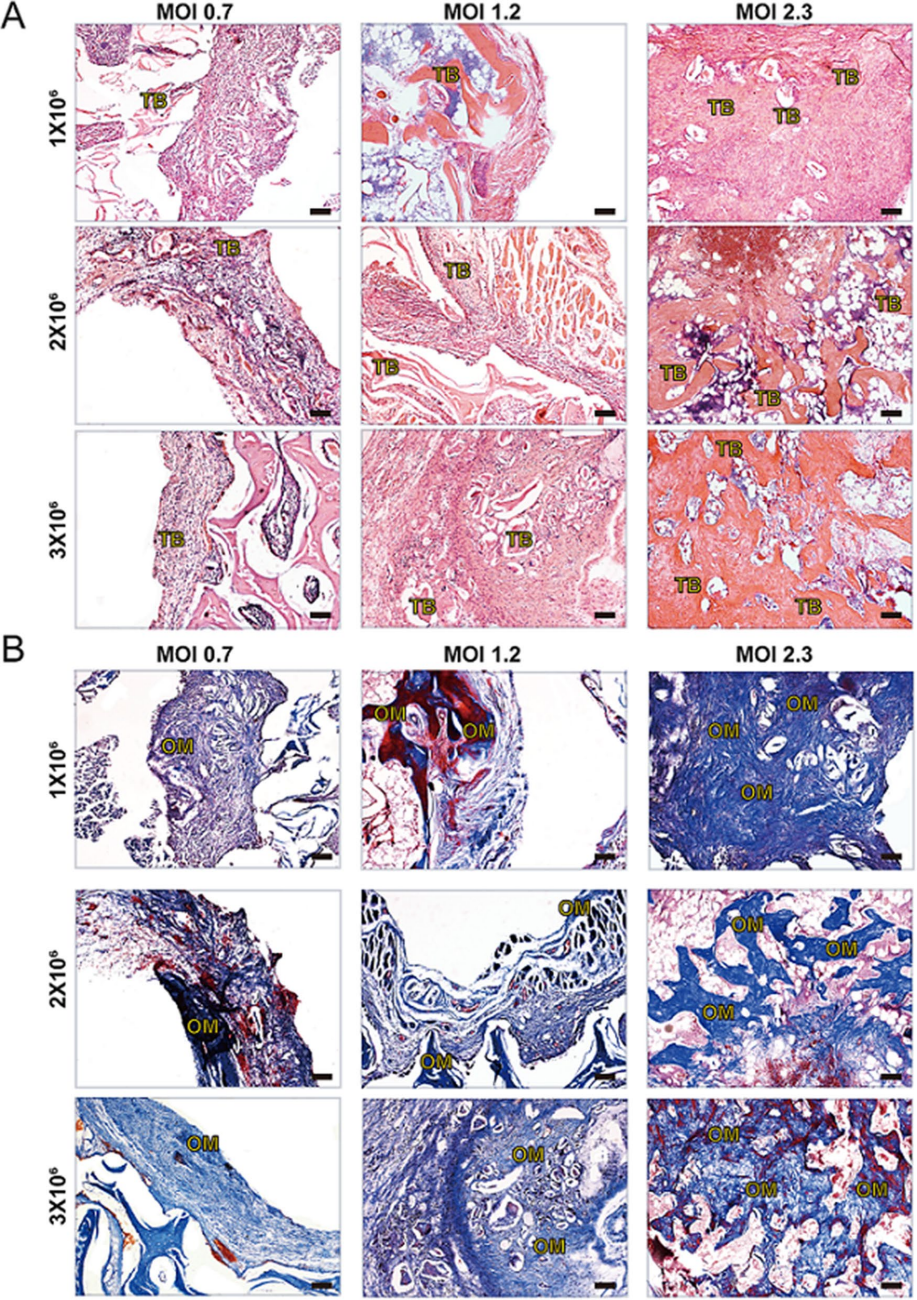
Histological analysis of the newly formed tissues with different cell concentrations and distinct BMP9 MOIs. After μCT imaging was completed, the samples were decalcified and subjected to paraffin-embedded sectioning for histologic evaluation, including **A** H&E staining and **B** Masson staining. The representative images show fields of new tissue formation inside the DBM constructs. TB, trabecular bone; OM, osteoid matrix. Scale bar = 100 μm

**Fig. 8:**
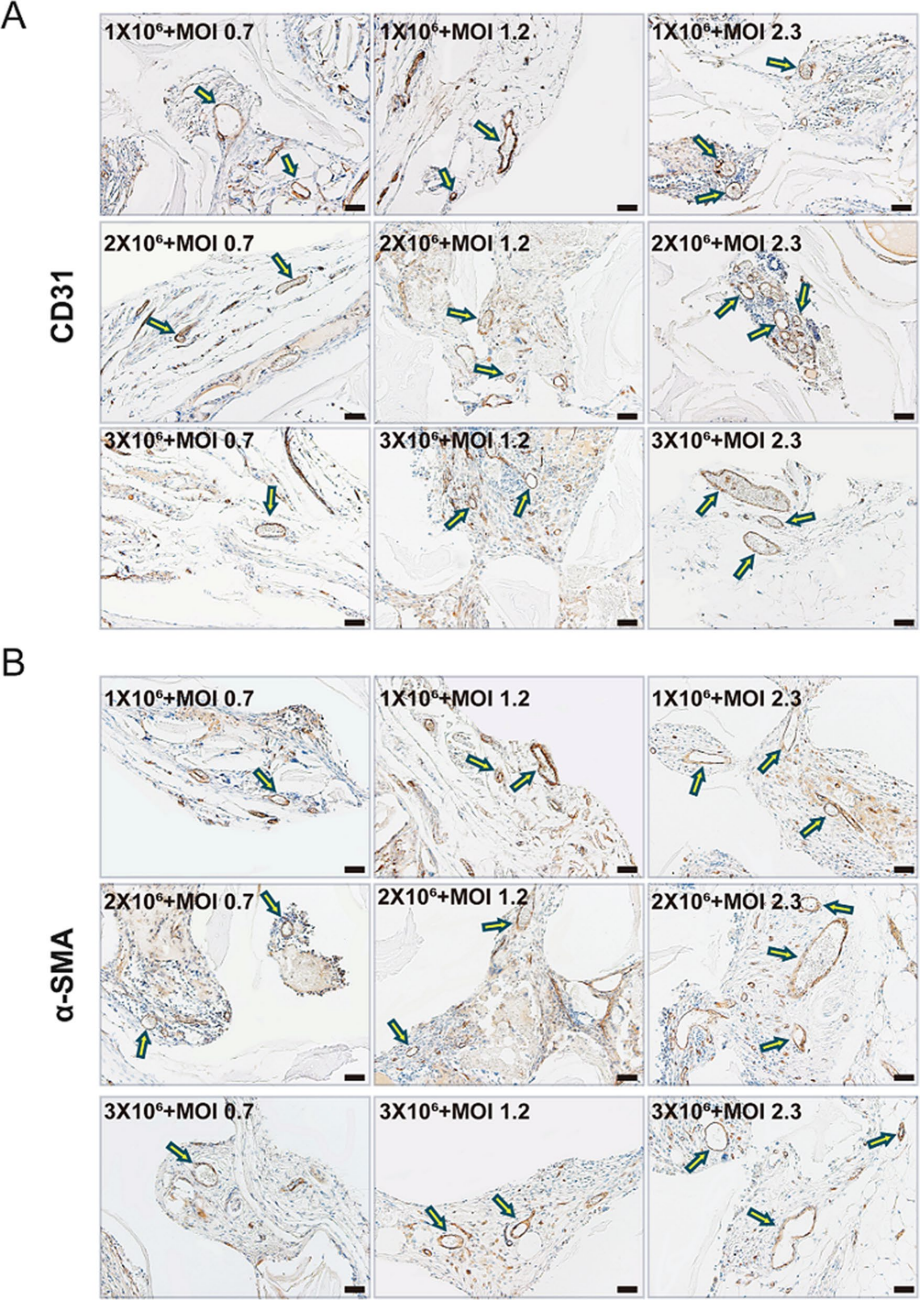
Immunohistochemical staining of the newly formed tissues with different cell concentrations and distinct BMP9 MOIs. **A** Representative immunohistochemical staining images of CD31 and **B** α-SMA. The newly formed blood vessels are indicated with arrows. Scale bar = 50 μm

Histologically, H&E staining analysis revealed that the DBM constructs injected with BMP9-stimulated PDLSCs formed evident trabecular bone in all groups, while the significant augmentation of newly formed bone correlated with the increased MOIs of BMP9, and the 1 × 10^6^ cells/construct under BMP9 stimulation at an MOI of 2.3 were adequate to form new bone tissue, nearly filling the whole defect area of DBM constructs (Fig. 7A). Masson trichrome staining analysis further confirmed that the DBM constructs injected with 1 × 10^6^ PDLSCs under BMP9 stimulation at an MOI of 2.3 could form apparently mature and well-mineralized bony masses (Fig. 7B). The immunohistochemical staining assays revealed the expression of osteogenic marker osteocalcin (OCN), which was also in consistent with the results (Additional file 1: Fig. S5A, B). Moreover, the presence of newly formed blood vessels was further verified, as the expression of CD31 and α-SMA was detected in the regenerative tissues by immunohistochemical staining (Fig. 8A, B). Similar to the H&E staining and Masson trichrome staining analyses, the enhanced positive staining of CD31 and α-SMA correlated with the increased BMP9 MOIs, consistent with the fact that BMP9 has both osteogenic and angiogenic activities [46]. In addition, compared to cell doses at 2 × 10^6^/construct and 3 × 10^6^/construct, 1 × 10^6^/construct was adequate to promote efficacious new blood vessel formation. These results demonstrated that an optimal dose of PDLSCs under a specific extent of BMP9 stimulation was efficacious for both new bone and new blood vessel formation in DBM constructs.

## Discussion

In the present study, we prepared the DBM from porcine lumbar vertebrae bone and fabricated it into a cylinder-shaped construct to be the modified ectopic system, as well as the ectopic intrabony defect repair model. Then, we explored the suitable stem cell sources among three types of hDMSCs and optimized the combination of PDLSCs and BMP9 for efficacious osteogenic regeneration based on this ectopic intrabony defect model. We found that BMP9-stimulated PDLSCs yielded the most robust new bone formation among the three types of hDMSCs, and an optimal dose of PDLSCs under a specific extent of BMP9 stimulation was adequate to promote efficacious new bone and new blood vessel formation in DBM constructs.

Traditionally, bone healing has been studied based on the construction of orthotopic bone defects [47], while the construction of orthotopic bone defects is indeed laborious and costly if not possible. Compared to the common orthotopic locations at the calvarium, radius and the femur, the bone defect at maxillofacial sites could be more difficult and required more elaborate surgical procedures. Thus, inspired from the orthotopic maxillofacial intrabony defects, mimicking these orthotopic intrabony defects into a simple and effective model to evaluate various candidates for bone regeneration should be meaningful, which could also reduce the number of animals used and improve welfare standards in the future studies. Besides, the ectopic system is widely used for the evaluation of new bone formation in tissue engineering therapies as an alternative, which is easily handled with the price of lacking a bony environment [10]. To solve these conflicts, we modified the ectopic system with the use of DBM to develop a container-like construct, which could be an ectopic intrabony defect model and enable us to easily inject the repair materials into the construct. As a commonly used material, DBM has been previously investigated to supply a microenvironment of bone tissue containing collagen (mainly type I), ECM proteins and growth factors, including bone morphogenetic proteins (BMPs) and vascular endothelial growth factor (VEGF) [12]. In addition, DBM-based materials have been demonstrated to be chemotactic for stem/progenitor cells and are supposed to be beneficial to endogenous stem cell recruitment, similar to the in vivo bone defect environment [48, 49]. Thus, DBM can provide an inductive bony environment for new bone and new vasculum regeneration [50]. Our micro-CT images showed the formation of a large amount of new bone in the DBM construct containing PBS without cells. This result confirmed the osteoconduction and osteoinduction of our designed DBM construct. Moreover, to recapitulate the traditional ectopic osteogenesis procedure, we subcutaneously implanted plain DBM constructs (without cells) into athymic nude mice in advance and then injected the cell suspension into the central area of the construct 7 days later. In this way, we facilitated the generation of vascularized tissue utilizing the in vivo environment as a bioreactor to enable the internal environment of the DBM constructs to mimic the orthotopic systems [51–53].

Several species and sources have been used to prepare DBM, including bovine femurs, bovine tibiae, goat articular cartilage and human epiphyseal bone [54, 55]. In this article, we further optimized DBM constructs prepared from porcine L4–L5 vertebrae, which have been demonstrated to be the most similar to that of humans and feel the same as living people [56]. The part of cancellous bone in porcine lumbar vertebrae was selected; in that cancellous bone is reported to be the position at which 80% of bone remodeling processes occur [57]. The cancellous part of the porcine lumbar vertebrae was confirmed to possess a uniformly trabecular bone structure under micro-CT analysis, which allowed the DBM to construct optimal properties of naturally open and interconnected porous structures to promote bone and vessel formation.

The key points in the development of novel tissue engineering therapy are to determine the suitable sources and doses of seed cells needed [58]. For the regeneration of maxillofacial bone defect, PDLSCs, DPSCs and SCAPs could be perfectly efficient candidates and are popularly studied hDMSCs that possess various differential abilities [59–63]. All three hDMSCs have been analyzed for osteogenic differentiation. Our qPCR results revealed that these three hDMSCs could all response to the BMP9-mediated osteogenic signaling. However, the comparison of the osteogenic activity among them was doubtful in the reported literature. Hu L et al. found that DPSCs, PDLSCs and SCAP cell sheets have similar characteristics in vitro, but their osteogenic and angiogenic characteristics are different in vivo [64]. Moreover, other previous studies have revealed that PDLSCs exhibit stronger osteogenic activity than DPSCs in in vitro systems [14]. Similarly, in the present study, the results of BMP9-stimulated PDLSCs yielding the most robust new bone formation among the three hDMSCs in the modified ectopic system should be reasonable.

Another highlight of this study is that the DBM constructs can also be used as repeatable and controlled intrabony defects to screen for appropriate doses of seed cells under specific extents of biofactor stimulation. We demonstrated that PDLSCs with different doses of cell numbers varying from 1 × 10^6^/construct to 3 × 10^6^/construct under BMP9 stimulation at an MOI of 2.3 could nearly repair the whole defect area inside the DBM construct and even exceed the height of the construct. In addition, histological staining revealed that these groups could all generate many new blood vessels and bone tissues both inside and outside the construct, which demonstrated the great integrative repair capacity of the regenerative organization to the DBM construct. These results suggested that an optimal number of seed cells under a specific extent of BMP9 stimulation is enough to repair critical-sized bone defects. In MSC therapy, the seed cell sources and sub-optimal doses usually exhibit inconsistent outcomes in distinct clinical trials [65]. Meanwhile, MSC treatment is costly, and the greater the demand for seed cells, the higher the price. Therefore, exploration of the appropriate MSC source and dose under a certain extent of biological factor stimulation for the repair of specific-sized defects is essential in engineering therapy. Collectively, the DBM constructs designed in our study can be used not only as a modified ectopic system but also as a novel ectopic critical-sized intrabony defect to optimize the suitable dose of seed cells, which is enough for the bone healing of specific-sized defects. The size of the DBM constructs can be easily adjusted according to the demand. However, due to the objective of our study, mechanical force, which was significant for bone formation, was left out. Since the DBM constructs contained BMP9-stimulated hPDLSCs exhibited robust osteogenic activity, we will try to develop them for orthotopic maxillofacial defects repair in the next phase of our investigation. In addition, popular and effective strategies, such as the use of electrospinning [12] and hydrogels, can also be utilized to further modify DBM constructs in future research.

## Conclusions

In summary, the present study successfully fabricated a cylinder-shaped DBM construct from porcine lumbar vertebrae to modify the ectopic system and mimic the orthotopic maxillofacial intrabony defect. The use of constructs in optimizing the combination of suitable hDMSCs and BMP9 for intrabony defect repair revealed that BMP9-stimulated PDLSCs exhibited the most effective bone regeneration among the three hDMSCs, while an optimal dose of PDLSCs under a specific extent of BMP9 stimulation was adequate for new bone formation, excellent osseointegration and vascularization in DBM constructs. Therefore, using the modified ectopic system to further explore the optimized combination of suitable seed cells and biological factors for bone defect repair could provide significant new insights to develop efficacious bone tissue engineering therapies.

## Supplementary Information

**Additional file 1. Fig. S1**: The effect of BMP9 on the osteogenic differentiation ability of hDMSCs in vitro. (A) BMP9 stimulated the expression of early responsive target genes in hDMSCs. Human DPSCs, SCAPs and PDLSCs were infected with Ad-GFP and Ad-BMP9 for 24h. The expression levels of ID1, ID2 and CTGF in different groups were evaluated by qPCR. (B) BMP9 stimulated the expression of osteogenic genes in hDMSCs. The expression levels of RUNX2, OPN, ALP and OSX in different groups were evaluated by qPCR on days 3 and 7. The assays were carried out in three independent experiments. All data are the means ± SDs; *P<0.05 and **P<0.01. **Fig. S2**: Reconstructed 3D micro-CT images showing the heights of new bone formation. Representative images are shown. **Fig. S3**: The heights of new bone were analyzed using Mimics Research 19.0 software. All values are the means ± SDs; *P < 0.05, **P < 0.01, ***P < 0.001. **Fig. S4**: Reconstructed 3D micro-CT images of the whole constructs. The yellow regions represent the plain DBM constructs, while the blue regions are indicative of newly formed bony masses. Representative images are shown. **Fig. S5**: Immunohistochemical staining of osteogenic marker in newly formed tissues. (A) Representative immunohistochemical staining images of osteocalcin (OCN). Scale bar=50 μm. (B) The OCN positive cells were quantified by IOD (Integrated Optical Density) with Image-Pro Plus. All values are the means ± SDs; ***P < 0.001, # no statistical significance. **Table S1**: Primer sequences.

## Data Availability

The data that support the findings of this study are available from the corresponding author upon reasonable request.

## Abbreviations

DBM: Decellularized bone matrix
hDMSCs: Human dental mesenchymal stem cells
DPSCs: Dental pulp stem cells
PDLSCs: Periodontal ligament stem cells
SCAPs: Apical papillary stem cells
BMP9: Bone morphogenetic protein 9
rsDBM: Ring-shaped decellularized and demineralized bone matrix
Ad-BMP9: Adenoviruses expressing BMP9
Ad-GFP: Adenoviruses green fluorescent protein
MOI: Multiplicity of infection
BV/TV: Bone volume fraction
HU: Hounsfield units
H&E: Hematoxylin and eosin
VEGF: Vascular endothelial growth factor
FITC: Fluorescein isothiocyanate
PBS: Phosphate-buffered saline
2D: Two-dimensional
3D: Three-dimensional
RT-qPCR: Real-time quantitative polymerase chain reaction
cDNA: Complementary DNA
GAPDH: Glyceraldehyde-3-phosphate dehydrogenase
ID1: Inhibitor of differentiation 1
ID2: Inhibitor of differentiation 2
CTGF: Connective tissue growth factor
RUNX2: Runt-related transcription factor 2
OPN: Osteopontin
ALP: Alkaline phosphatase
OSX: Osterix
SPF: Specific pathogen free
OCN: Osteocalcin
DAB: Diaminobenzidine
IOD: Integrated optical density
