# Supplementary material for: Efficient bone regeneration of BMP9-stimulated human periodontal ligament stem cells (hPDLSCs) in decellularized bone matrix (DBM) constructs to model maxillofacial intrabony defect repair
Source: Stem Cell Res Ther. 2022 Dec 27;13:535. doi: 10.1186/s13287-022-03221-3 (PMC9795631; doi:10.1186/s13287-022-03221-3)
Supplement: Supplementary file 1 — Additional file 1. Fig. S1: The effect of BMP9 on the osteogenic differentiation ability of hDMSCs in vitro. (A) BMP9 stimulated the expression of early responsive target genes in hDMSCs. Human DPSCs, SCAPs and PDLSCs were infected with Ad-GFP and Ad-BMP9 for 24h. The expression levels of ID1, ID2 and CTGF in different groups were evaluated by qPCR. (B) BMP9 stimulated the expression of osteogenic genes in hDMSCs. The expression levels of RUNX2, OPN, ALP and OSX in different groups were evaluated by qPCR on days 3 and 7. The assays were carried out in three independent experiments. All data are the means ± SDs; *P<0.05 and **P<0.01. Fig. S2: Reconstructed 3D micro-CT images showing the heights of new bone formation. Representative images are shown. Fig. S3: The heights of new bone were analyzed using Mimics Research 19.0 software. All values are the means ± SDs; *P < 0.05, **P < 0.01, ***P < 0.001. Fig. S4: Reconstructed 3D micro-CT images of the whole constructs. The yellow regions represent the plain DBM constructs, while the blue regions are indicative of newly formed bony masses. Representative images are shown. Fig. S5: Immunohistochemical staining of osteogenic marker in newly formed tissues. (A) Representative immunohistochemical staining images of osteocalcin (OCN). Scale bar=50 μm. (B) The OCN positive cells were quantified by IOD (Integrated Optical Density) with Image-Pro Plus. All values are the means ± SDs; ***P < 0.001, # no statistical significance. Table S1: Primer sequences. [file 13287_2022_3221_MOESM1_ESM.docx]

**Stem Cell Research & Therapy**

**Supplementary information**

**Efficient bone regeneration of BMP9-stimulated human periodontal ligament stem cells (hPDLSCs) in** **decellularized bone matrix (DBM) constructs to model maxillofacial intrabony defect repair**

**Running Title: BMP9-stimulated hPDLSCs for intrabony defect repair**

Yuxin Zhang^1,2^, Wenping Luo^1,3^, Liwen Zheng^1,2^, Jing Hu^1,2^, Li Nie^1,3^, Huan Zeng^1,2^, Xi Tan^1,2^, Yucan Jiang^1,2^, Yeming Li^1,2^, Tianyu Zhao^1,3^, Zhuohui Yang^1,2^, Tong-Chuan He^4^**,** Hongmei Zhang^1,2^*

^1^ Chongqing Key Laboratory for Oral Diseases and Biomedical Sciences, the Affiliated Hospital of Stomatology of Chongqing Medical University, Chongqing, China

2Department of Pediatric Dentistry, The Affiliated Hospital of Stomatology,

Chongqing Medical University, Chongqing, China

3 Chongqing Municipal Key Laboratory of Oral Biomedical Engineering of Higher Education, Chongqing, China

4Molecular Oncology Laboratory, Department of Orthopaedic Surgery and Rehabilitation Medicine, The University of Chicago Medical Center, Chicago, IL 60637, USA

* Corresponding author

**Correspondence**

Hongmei Zhang, DDS, PhD

Chongqing Key Laboratory for Oral Diseases and Biomedical Sciences

The Affiliated Hospital of Stomatology

Chongqing Medical University

426 Songshibei Road, Chongqing 401147, China
Email: hmzhang@hospital.cqmu.edu.cn

**Figure S1. The effect of BMP9 on the osteogenic differentiation ability of hDMSCs in vitro.** (A) BMP9 stimulated the expression of early responsive target genes in hDMSCs. Human DPSCs, SCAPs and PDLSCs were infected with Ad-GFP and Ad-BMP9 for 24h. The expression levels of ID1, ID2 and CTGF in different groups were evaluated by qPCR. (B) BMP9 stimulated the expression of osteogenic genes in hDMSCs. The expression levels of Runx2, OPN, ALP and OSX in different groups were evaluated by qPCR on days 3 and 7. The assays were carried out in three independent experiments. All data are the means ± SDs; *P<0.05 and **P<0.01.


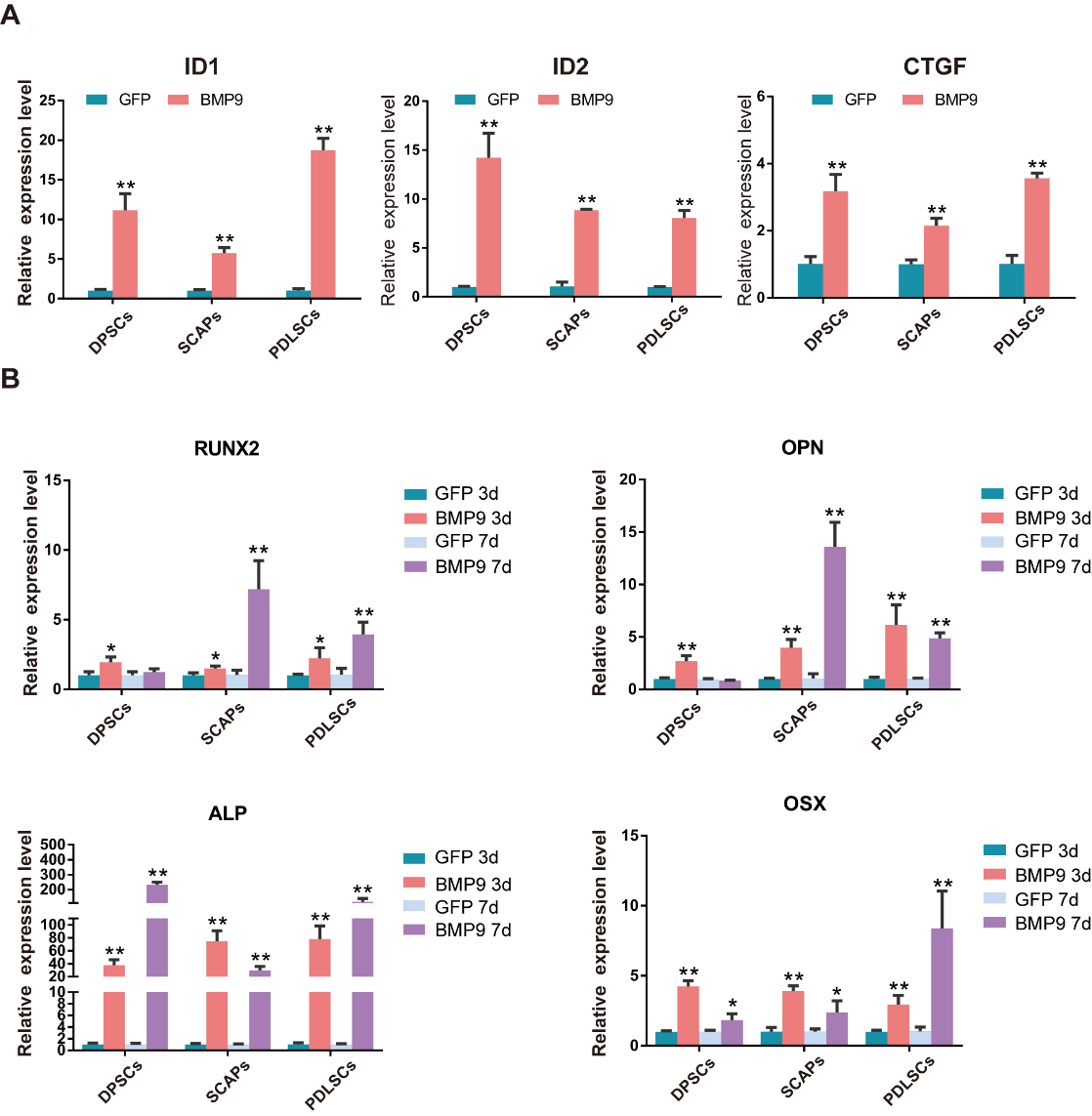


**Figure S2**: Reconstructed 3D micro-CT images showing the heights of new bone formation. Representative images are shown.


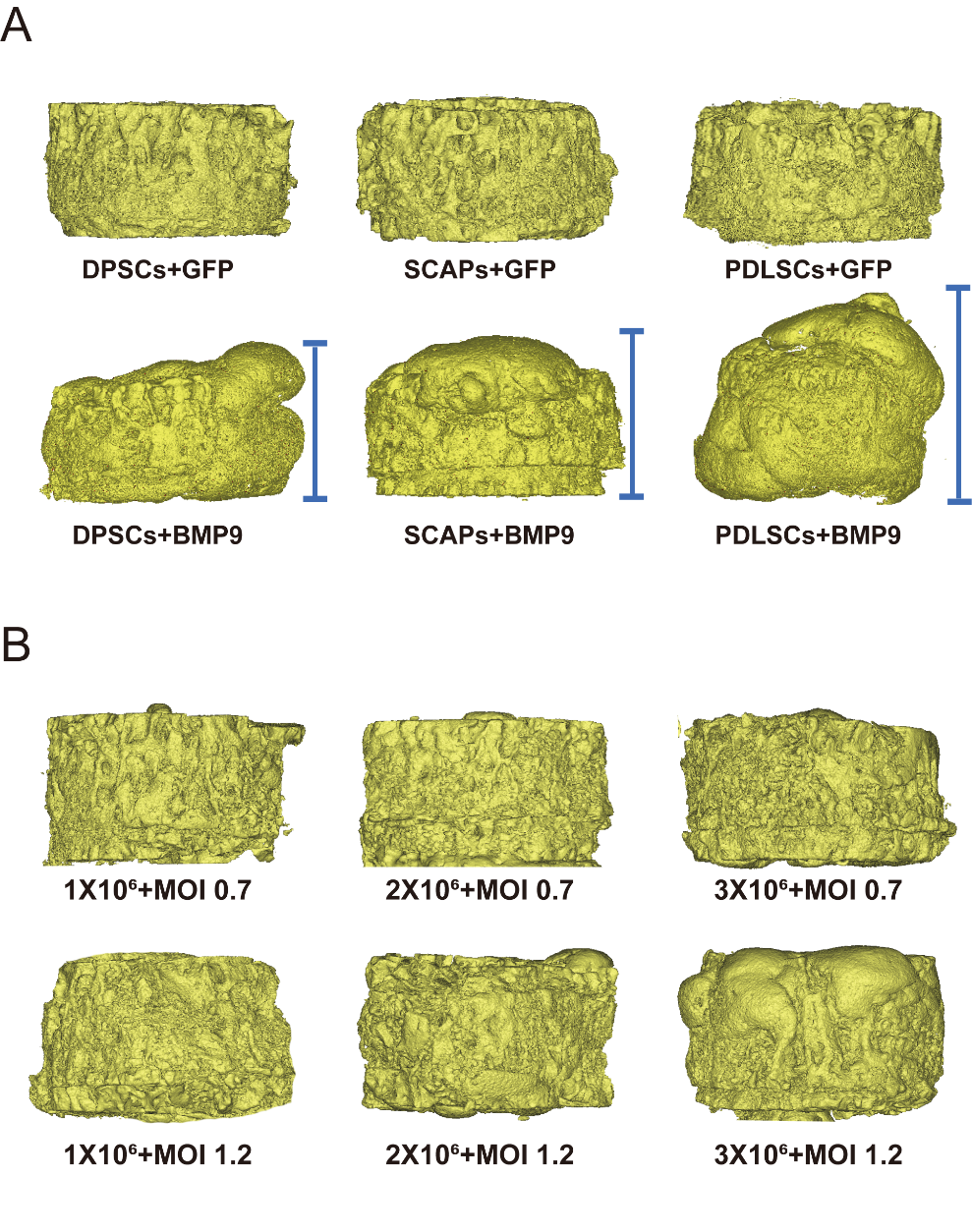


**Figure S3:** The heights of new bone were analyzed using Mimics Research 19.0 software. All values are the means ± SDs; *P < 0.05, **P < 0.01, ***P < 0.001.


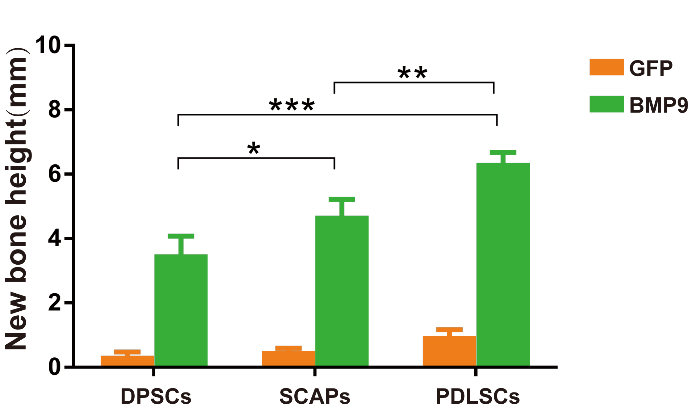


**Figure S4:** Reconstructed 3D micro-CT images of the whole constructs. The yellow regions represent the plain DBM constructs, while the blue regions are indicative of newly formed bony masses. Representative images are shown.


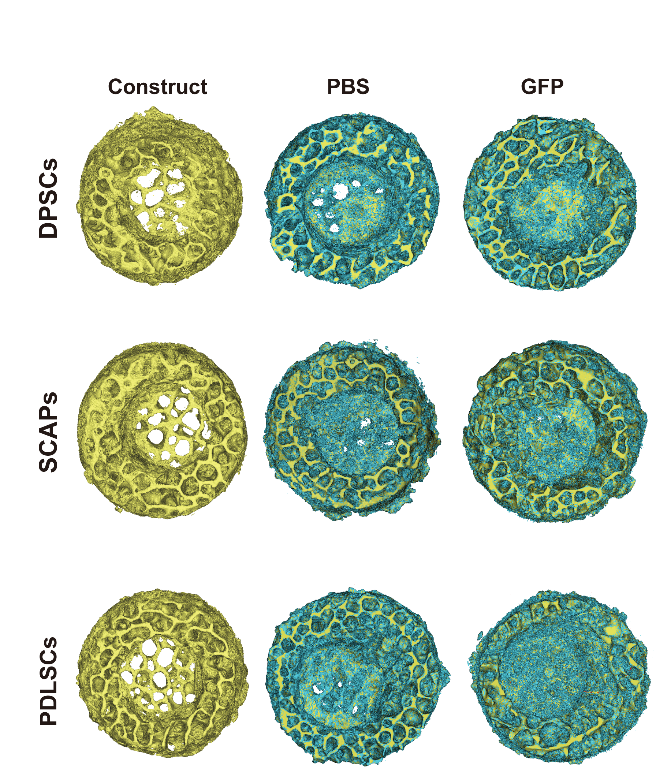


**Figure S5. Immunohistochemical staining of osteogenic marker in newly formed tissues.** (A) Representative immunohistochemical staining images of osteocalcin (OCN). Scale bar=50 μm. (B) The OCN positive cells were quantified by IOD (Integrated Optical Density) with Image-Pro Plus. All values are the means ± SDs; ***P < 0.001, # no statistical significance.


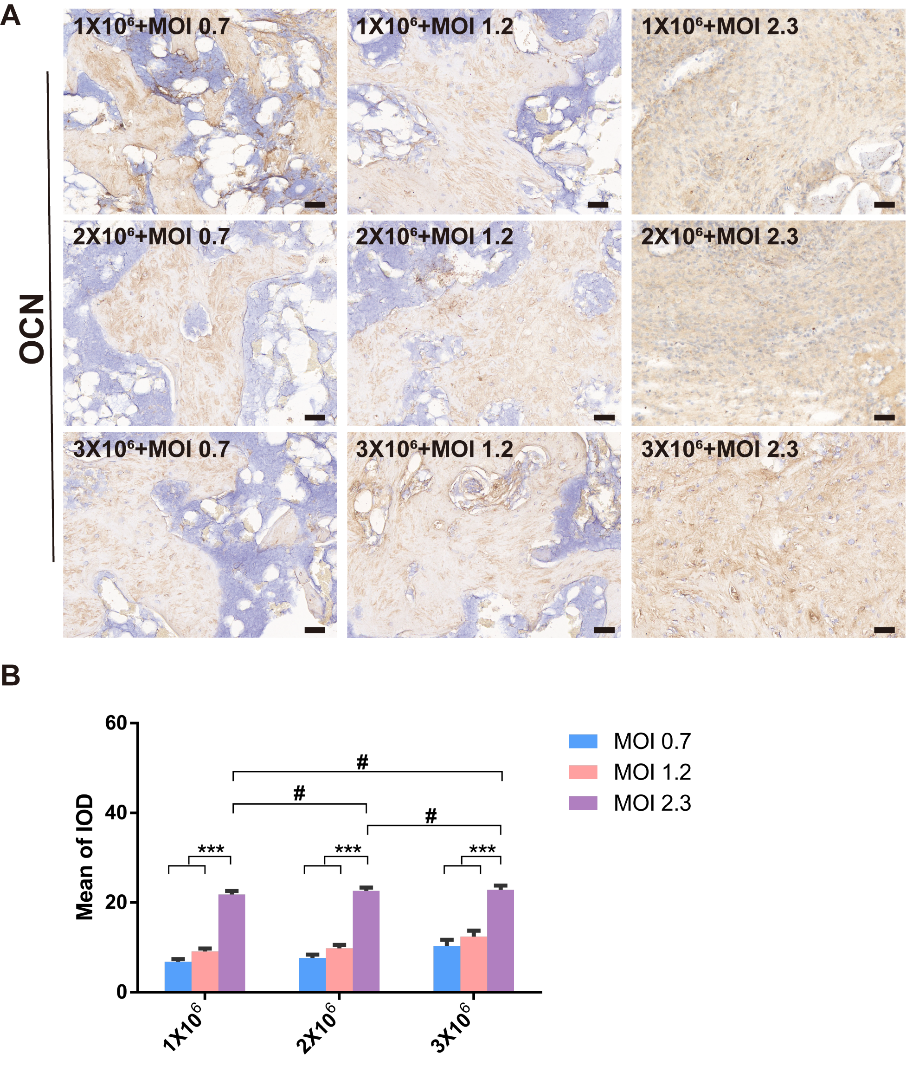


Table S1. Primer sequences.

| Gene | Forward | Reverse |
| --- | --- | --- |
| GAPDH | CGACCACTTTGTCAAGCTCA | GGTGGTCCAGGGGTCTTACT |
| ID1 | GGACGATCGCATCTTGTGTC | ATCGGTCTTGTTCTCCCTCA |
| ID2 | TATTGTCAGCCTGCATCACC | CACACAGTGCTTTGCTGTCA |
| CTGF | CCTGCAGGCTAGAGAAGCAG | TGGAGATTTTGGGAGTACGG |
| RUNX2 | CCCAGTATGAGAGTAGGTGTCC | GGGTAAGACTGGTCATAGGACC |
| OPN | CGAGGTGATAGTGTGGTTTATGG | GCACCATTCAACTCCTCGCTTTC |
| ALP | GCTGTAAGGACATCGCCTACCA | CCTGGCTTTCTCGTCACTCTCA |
| OSX | TTCTGCGGCAAGAGGTTCACTC | GTGTTTGCTCAGGTGGTCGCTT |
